# Egyptian hemodialysis patients' willingness to receive the COVID-19 vaccine booster dose: a multicenter survey

**DOI:** 10.1007/s40620-023-01586-z

**Published:** 2023-03-02

**Authors:** Hoda Mahmoud Mohammad Abdulaziz, Marwa Ahmed Saleh, Mohamed Essam Elrggal, Mariam E. Omar, Shymaa A. Hawash, Ahmed Mohamed Naguib Attiya, Karem Mohamed Salem, Alaa Abdel-Aziz Sabry

**Affiliations:** 1 Mansoura Nephrology and Dialysis Unit (MNDU), Department of Internal Medicine, Faculty of Medicine, Mansoura University, Mansoura, Egypt; 2 Kidney and Urology Center, Alexandria, Egypt; 3 Faculty of Medicine, Alexandria Main University Hospital (AMUH), Alexandria University, Alexandria, Egypt; 4 Alexandria University Hospitals, Alexandria, Egypt; 5 Urology and Nephrology Center, Mansoura University, Mansoura, Egypt; 6 Department of Internal Medicine, Faculty of Medicine, Fayoum University, Fayoum, Egypt; 7 Nephrology department, AlQabbary Specialty Hospital, Alexandria, Egypt

**Keywords:** COVID-19 vaccine, Booster dose, Vaccine hesitancy, SARS-CoV-2, Hemodialysis

## Abstract

**Background:**

Health authorities have struggled to increase vaccination uptake since the COVID-19 vaccines became available. However, there have been increasing concerns about declining immunity after the initial COVID-19 vaccination with the emergence of new variants. Booster doses were implemented as a complementary policy to increase protection against COVID-19. Egyptian hemodialysis (HD) patients have shown a high rate of hesitancy to COVID-19 primary vaccination, yet their willingness to receive booster doses is unknown. This study aimed to assess COVID-19 vaccine booster hesitancy and its associated factors in Egyptian HD patients.

**Methods:**

A face-to-face interview was conducted with closed-ended questionnaires distributed to healthcare workers in seven Egyptian HD centers, mainly located in three Egyptian governorates, between the 7th of  March and the 7th of April 2022.

**Results:**

Among 691 chronic HD patients, 49.3% (*n* = 341) were willing to take the booster dose. The main reason for booster hesitancy was the opinion that a booster dose is unnecessary (*n* = 83, 44.9%). Booster vaccine hesitancy was associated with female gender, younger age, being single, Alexandria and urban residency, the use of a tunneled dialysis catheter, not being fully vaccinated against COVID-19. Odds of booster hesitancy were higher among participants who did not receive full COVID-19 vaccination and among those who were not planning to take the influenza vaccine (10.8 and 4.2, respectively).

**Conclusion:**

COVID-19 booster-dose hesitancy among HD patients in Egypt represents a major concern, is associated with vaccine hesitancy with respect to other vaccines and emphasizes the need to develop effective strategies to increase vaccine uptake.

**Graphical abstract:**

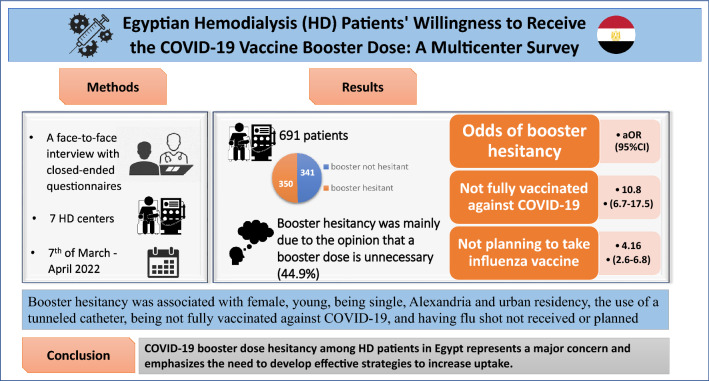

**Supplementary Information:**

The online version contains supplementary material available at 10.1007/s40620-023-01586-z.

## Introduction

On the 11th March, 2020, coronavirus disease 2019 (COVID-19), the disease caused by severe acute respiratory syndrome coronavirus 2 (SARS-CoV-2), was declared a pandemic by The World Health Organization (WHO) [1]. To contain the pandemic, several vaccines were rapidly developed, tested, and made available for utilization. The Food and Drug Administration (FDA) granted an Emergency Use Authorization to the first COVID-19 vaccine on 11th December, 2020 [2]. Since then, vaccination efforts have been at the forefront of measures to bring the pandemic to an end and minimize COVID-19 related morbidity and mortality. However, low vaccine uptake impedes efforts to contain the pandemic. In 2019, vaccine hesitancy was identified by the WHO as one of the ten most dangerous threats to worldwide health. Vaccine hesitancy is defined as a delay in acceptance or refusal of vaccines despite availability of vaccine services [3]. It is context-dependent and varies across time, place, and different vaccines, and is influenced by factors such as confidence, satisfaction, accessibility, and sociodemographic characteristics [4]. Hesitancy to vaccination may also be linked to the online spread of misinformation and conspiracy assumptions, often through social media [5, 6]. Moreover, there had been increasing concerns of declining immunity from the initial COVID-19 dose in the light of emerging new variants. Booster doses were then recommended to increase protection against COVID-19 [7].

Patients on maintenance hemodialysis (HD) have a substantial risk of developing COVID-19. Such patients on HD typically have several medical comorbidities, including diabetes mellitus, obesity, and compromised immune system, all of which increase their risk of developing devastating COVID-19–related complications. One study found that the estimated 90-day mortality among COVID-19-infected HD patients exceeds 20% in the United States [8]. There is evidence from China and Italy showing that patients on maintenance HD with COVID-19 suffer higher mortality and more severe disease than the general population [9–11]. Following the authorization of emergency use of SARS-CoV-2 vaccines, their uptake among HD patients became crucial to mitigating poor outcomes of COVID-19 observed in the dialysis population. COVID-19 vaccines are highly effective at reducing severe illness and death from COVID-19, and moreover, vaccination is safe, with low risks of severe adverse effects [12].

Nevertheless, several studies showed that a considerable proportion of patients on dialysis were reluctant to take the COVID-19 vaccines [13–15]. Various reasons have been identified as being associated with COVID-19 vaccine hesitancy in HD patients [16]. In Egypt, the Egyptian government made vaccination against COVID-19 mandatory and since then the rate of vaccination has been increasing. Nevertheless, little is known about the uptake of the booster dose in patients on HD. We therefore set out to assess the willingness of Egyptian patients on HD to receive the COVID-19 booster dose.

## Methods

### Study design and participants

This cross-sectional study recruited 691 chronic HD patients aged 18 years or above from seven different HD centers distributed mainly in three Egyptian governorates; Dakahlia**,** Alexandria, and Fayoum. HD patients with dementia and those with language barriers that would impair basic comprehension of the questions were excluded. After obtaining informed consent from eligible patients, a healthcare worker belonging to each HD unit explained the aim of the questionnaire to each patient and fulfilled the related questions. The study was conducted between the 7th of both March and April 2022.

### Sample size justification

G*Power software was used for power analysis to enable sample size calculation in order to detect hypothesized effect [17, 18]. Using the Cohen’s benchmarks of moderate effect sizes (0.3) for Chi-square, alpha level 5%, and 95% power, the minimum sample size required was 220. The sample size was increased to be more representative.

### Survey

The survey consisted of 27 questions with three major subheadings: demographic data (7 items), COVID-19 vaccine (10 items), and health of the responder and family (10 items). These subheadings followed the conceptual framework recommended by the Strategic Advisory Group of Experts on Immunization (SAGE) Working Group on Vaccine Hesitancy [19]. The majority of the survey questions were drawn from published surveys [13, 20, 21]. Survey questions were proposed by health care workers within each dialysis unit.

### Ethical considerations

The study protocol was reviewed and approved by the Mansoura University institutional research board with approval number: R.22.03.1639.

### Statistical analysis

The collected data were coded, processed and analyzed using Statistical Package for the Social Sciences (SPSS) program for Windows. Qualitative data were represented as frequencies and relative percentages. Chi-Square test was used to test the association between categorical variables. Binary logistic regression was utilized to test the association between independent and dependent variables. Subsequently, multivariate logistic regression was performed to compute the adjusted odds ratio (AOR) and 95% confidence interval (95% CI) for predictors of booster hesitancy. *P* value < 0.05 was considered significant.

## Results

### Demographic characteristics

**Fig. 1:**
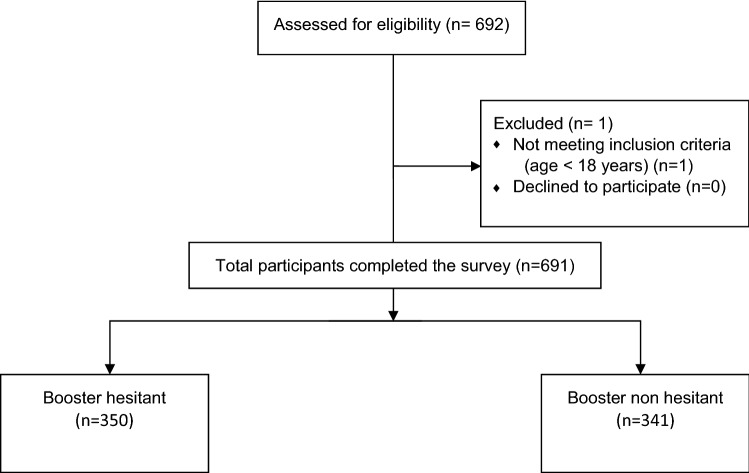
Study flow chart

Among 692 chronic HD patients assessed for eligibility, 691 participants completed the survey (Fig. 1). The majority of patients were above 50 years old, married, living in urban areas in a multi-generational household, not working/retired, not diabetic, and on HD for more than 3 years (Table 1).

**Table 1:** Clinical and demographic characteristics of the study participants

Characteristic	*N*	%
Gender		
Male	407	58.9
Female	284	41.1
Age (years)		
18–30	93	13.5
31–50	257	37.2
> 50	341	49.3
Marital status		
Single	137	19.8
Married	488	70.6
Divorced/widowed	66	9.6
Governorate		
Dakahlia	145	21
Alexandria	232	33.6
Fayoum	250	36.2
Others^a^	64	9.3
Education level		
Uneducated	80	11.6
Basic level	188	27.2
Secondary level	206	29.8
Tertiary level	217	31.4
Residence		
Rural	293	42.4
Urban	398	57.6
Working status		
Not working/retired	557	80.6
Working	134	19.4
Living in a multi-generational household	393	56.9
Diabetes	108	15.6
Previous renal transplantation	63	9.1
Immunosuppressive therapy in the last 12 months		
No	619	89.6
Yes	45	6.5
Don’t know	27	3.9
Dialysis via a tunneled catheter	53	7.7
Dialysis vintage		
< 1 year	116	16.8
1–3 years	146	21.1
> 3 years	429	62.1
Infection with SARS-CoV-2	189	27.4
Before vaccination	152	22.0
Between 1st and 2nd dose of vaccine^b^	17	2.5
After receiving all required doses^c^	20	2.9
Close family member with COVID-19	240	34.7
Close family member died of COVID-19	110	15.9

^a^Other governorates and number of participants were (Asyut 1, Damietta 1 Gharbia 42, Beheira 12, Kafr el-Sheikh 4, Monufia 3, Beni-Suef 1)

^b^Between first and second dose of vaccine (for those vaccinated with BioNTech/Pfizer, Moderna, AstraZeneca, Sinopharm, or Sinovac vaccines)

^c^After receiving all required doses (two doses of BioNTech/Pfizer, Moderna, AstraZeneca, Sinopharm, or Sinovac vaccines or a single dose of Janssen/Johnson & Johnson vaccine)

### COVID-19 vaccination status

Participants of this study received information regarding COVID-19 vaccines mainly from dialysis staff (*n* = 349, 50.5%), television (*n* = 212, 30.7%), treating physician (*n* = 191, 27.6%), and social media (*n* = 181, 26.2%). Few patients (*n* = 23, 3.3%) reported they had no information about COVID-19 vaccines. Of the studied patients, 486 (70.3%) were fully vaccinated against COVID-19. The most commonly received vaccine was Sinopharm (*n* = 167, 34.4%), while Janssen / Johnson & Johnson was the least received vaccine (*n* = 2, 0.4%). Table 2 summarizes the type of administered vaccine, the reasons for not receiving full COVID-19 vaccination, as well as the severity and fear of COVID-19 vaccine side effects.

**Table 2:** COVID-19 and Influenza vaccination status

Question	*N*	%
Fully vaccinated against COVID-19	486	70.3
Type of vaccine (*N* = 486)		
Don't know	79	16.3
BioNTech-Pfizer (2 doses)	49	10.1
Moderna (2 doses)	4	0.8
AstraZeneca (2 doses)	81	16.7
Sinopharm (2 doses)	167	34.4
Sinovac (2 doses)	104	21.4
Janssen/Johnson & Johnson (1 dose)	2	0.4
Reason for not receiving full vaccination (*N* = 205)		
Confidence	98	47.8
Complacency	32	15.6
Convenience/constraints	26	12.7
Calculation of risk	19	9.3
Collective responsibility	30	14.6
Severity of COVID-19 vaccine side effects (*N* = 486)		
No/negligible	325	66.9
Moderate	124	25.5
Very high	37	7.6
Level of fear accompanying the side effects of COVID-19 vaccine		
No/very low	328	67.5
Medium	128	26.3
Very high	30	6.2
Receipt of Influenza vaccine		
No	590	85.4
Yes	101	14.6
Planning to get the Influenza vaccine		
No	399	57.7
Yes	168	24.3
Have not decided	124	17.9

### Willingness to receive a COVID-19 vaccine booster dose

**Fig. 2:**
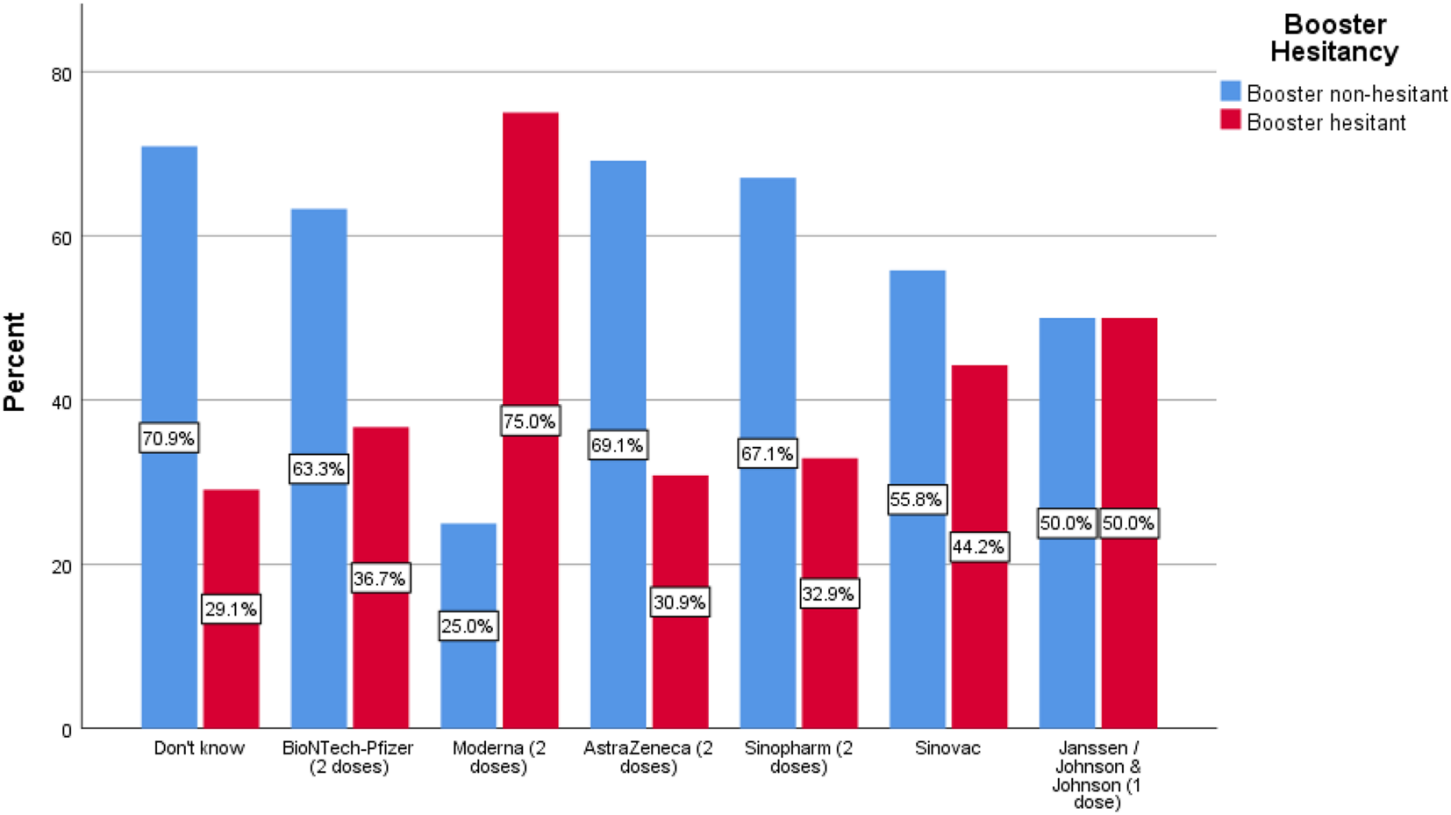
Frequency of booster hesitancy and non-hesitancy as regards the type of previous COVID-19 vaccines

Of 691 total participants, 341 (49.3%) were willing to take the booster dose, and the remaining 350 (50.7%) were booster-dose hesitant. The primary reason for hesitancy was the opinion that a booster dose is unnecessary (*n* = 83, 44.9%), followed by safety uncertainties (*n* = 72, 38.9%) and the side effects experienced after the previous doses (*n* = 30, 16.2%). Unwillingness to receive the booster dose of the COVID-19 vaccine was significantly higher among females, those aged 18–30 years versus age above 50 years, single individuals, Alexandria and urban residents, dialysis via tunneled catheter, those who are not fully vaccinated, and vaccinated patients with moderate to severe side effects (Table 3). The frequency of booster hesitancy and non-hesitancy as regards the type of previous COVID-19 vaccination is shown in Fig. 2.

**Table 3:** Differences between individuals with and without booster hesitancy

Parameters	Booster hesitant *N* = 350 *N* (%)	Booster non-hesitant *N* = 341 *N* (%)	*χ*^2^	*φ*/Cramer’s *V*	*p* value
Gender					
Male	190 (54.3%)	217 (63.6%)	6.238	− 0.095	**0.013**
Female	160 (45.7%)	124 (36.4%)			
Age					
18–30 years	67 (19.1%) a	26 (7.6%) b	22.074	0.179	** < 0.001**
31–50 years	131 (37.4%) a	126 (37%) a			
> 50 years	152 (43.4%) a	189 (55.4%) b			
Marital status					
Single	101 (28.9%) a	36 (10.6%) b	36.419	0.230	** < 0.001**
Married	220 (62.9%) a	268 (78.6%) b			
Divorced/widowed	29 (8.3%) a	37 (10.9%) a			
Governorate					
Dakahlia	68 (19.4%) a	77 (22.6%) a	40.203	0.241	** < 0.001**
Alexandria	151 (43.1%) a	81 (23.8%) b			
Fayoum	93 (26.6%) a	157 (46%) b			
Others	38 (10.9%) a	26 (7.6%) a			
Education level					
Uneducated	46 (13.1%)	34 (10%)	6.156	0.094	0.104
Primary level	85 (24.3%)	103 (30.2%)			
Secondary level	99 (28.3%)	107 (31.4%)			
Tertiary level	120 (34.3%)	97 (28.4%)			
Working status					
Not working	286 (81.7%)	271 (79.5%)	0.555	0.028	0.456
Working	64 (18.3%)	70 (20.5%)			
Residence					
Rural	132 (37.7%)	161 (47.2%)	6.382	− 0.096	**0.012**
Urban	218 (62.3%)	180 (52.8%)			
Previous COVID-19 infection	89 (25.4%)	100 (29.3%)	1.320	0.044	0.251
Not receiving vaccine information	15 (4.3%)	8 (2.3%)	2.020	− 0.054	0.155
Fully vaccinated	171 (48.9%)	315 (92.4%)	156.766	0.476	** < 0.001**
Severity of S/E (*N* = 486)					
No/minimal	94 (55%) a	231 (73.3%) b	16.884	0.186	** < 0.001**
Moderate	59 (34.5%) a	65 (20.6%) b			
Severe	18 (10.5%) a	19 (6%) a			
Diabetes mellitus	52 (14.9%)	56 (16.4%)	0.321	0.022	0.571
Previous renal transplantation	38 (10.9%)	25 (7.3%)	2.591	− 0.061	0.107
Dialysis via a tunneled catheter	35 (10%)	18 (5.3%)	5.437	− 0.089	**0.020**
Dialysis vintage					
< 1 year	59 (16.9%)	57 (16.7%)	0.031	0.007	0.984
1–3 years	73 (20.9%)	73 (21.4%)			
> 3 years	218 (62.3%)	211 (61.9%)			
Family member with COVID-19	123 (35.1%)	117 (34.3%)	0.053	− 0.009	0.818
Family member died of COVID-19	63 (18%)	47 (13.8%)	2.295	− 0.058	0.130
Multigenerational household	179 (51.1%)	214 (62.8%)	9.498	0.117	**0.002**
Flu shot taken	39 (11.1%)	62 (18.2%)	6.857	0.100	**0.009**
New flu shot planned					
No	244 (69.7%) a	155 (45.5%) b	64.060	0.304	** < 0.001**
Yes	41 (11.7%) a	127 (37.2%) b			
Have not decided	65 (18.6%) a	59 (17.3%) a			

Notes: For this analysis negative and “don’t know” responses were combined as “no” category. The test of significance is the Chi-Square test. Measures of the strength of association are *φ* (for 2×2 table) and Cramer’s V (for others). Z-tests for comparisons of column proportions (with Bonferroni adjustment of *p* values) are presented as letters (similar letters [a, a] = insignificant difference, while different letters [a, b] = significant difference

### Preferences of type of COVID-19 vaccine booster dose

**Fig. 3:**
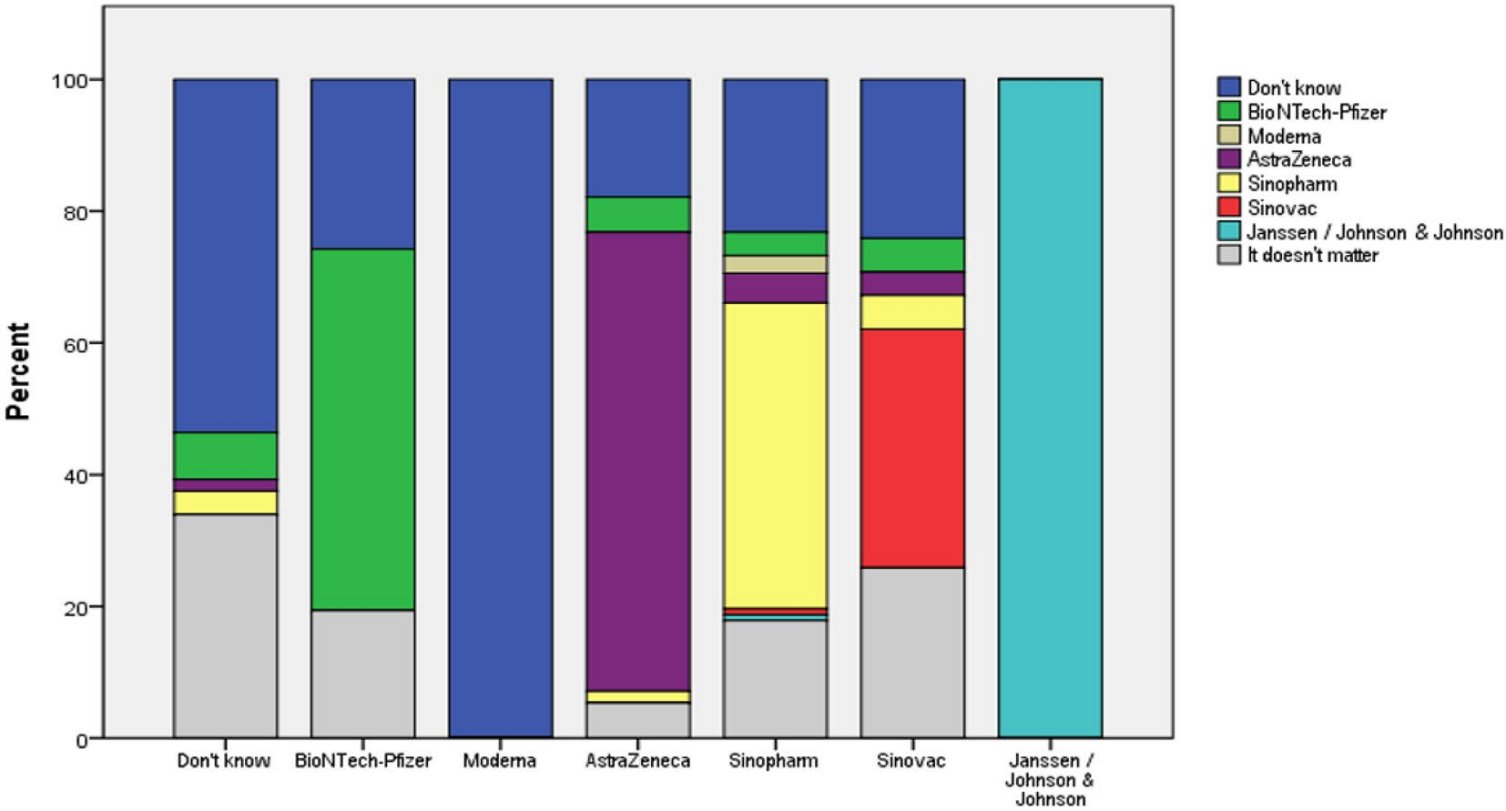
The preference of a specific COVID-19 vaccine to be used as the potential booster dose in previously vaccinated patients

Participants who were willing to receive a COVID-19 vaccine booster dose did not always prefer immunization with the same vaccine as administered previously. Figure 3 shows the preferences of surveyed individuals toward a particular COVID-19 vaccine they wished to receive as the potential booster dose.

In general, 29.6% (101) of patients willing to receive a COVID-19 vaccine booster dose could not decide at the moment of the survey which vaccine to take, while 20.2% (*n* = 69) had no preference with regard to a specific vaccine. However, most participants who completed their initial regimen with AstraZeneca and BioNTech-Pfizer wished to receive a potential booster dose provided by the same manufacturer (69.6%, *n* = 39 and 54.8%, *n* = 17, respectively). In the case of Sinopharm, 46.4% (*n* = 52) of surveyed individuals were interested in receiving it as a booster dose, while in the case of Sinovac, 36.2% (*n* = 21) of subjects. The only patient who initially received Janssen / Johnson & Johnson vaccine and was willing to receive a booster dose preferred to receive the same vaccine.

### Predictors of COVID-19 vaccine booster-dose hesitancy

Table 4 shows the results of binary logistic regression which was run to ascertain the effects of 10 variables on the likelihood of booster hesitancy. On univariable analysis, all 10 predictor variables were statistically significant. Accordingly, all were entered in a multivariable analysis model. The model was statistically significant (*χ*^2^ = 234.909, *P* < 0.001). The model correctly classified 71.8% of participants with 61.1% sensitivity and 82.7% specificity, and it explained 38.4% of the variance in booster hesitancy (Nagelkerke *R*^2^ = 0.384). Of the 10 predictor variables, only not being fully vaccinated and not planning a new flu shot were the two statistically significant independent predictors of the likelihood of booster hesitancy. Participants who did not receive full COVID-19 vaccination and those who were not planning to take the influenza vaccine had 10.8- and 4.2-times higher odds of exhibiting booster hesitancy, respectively.

**Table 4:** Predictors of the likelihood of Booster Hesitancy (*n* = 691)

Predictor	Univariable			Multivariable		
	COR	95% CI	*p* value	AOR	95% CI	*p* value
Gender			**0.013**			0.360
Male	r(1)	r(1)		r(1)	r(1)	
Female	1.47	1.09–2		1.19	0.82–1.71	
Age group						
18–30 years	r(1)	r(1)		r(1)	r(1)	
31–50 years	0.4	0.24–0.68	**0.001**	0.68	0.33–1.41	0.300
> 50 years	0.3	0.19–0.52	** < 0.001**	0.47	0.21–1.02	0.057
Marital status			** < 0.001**			0.190
Married/Divorced/Widowed	r(1)	r(1)		r(1)	r(1)	
Single	3.4	2.3–5.2		1.54	0.81–2.92	
Alexandria residence			** < 0.001**			0.429
No	r(1)	r(1)		r(1)	r(1)	
Yes	2.4	1.76–3.38		1.23	0.74–2.02	
Residence			**0.012**			0.108
Rural	r(1)	r(1)		r(1)	r(1)	
Urban	1.48	1.09–2		0.69	0.44–1.09	
Fully COVID-19 vaccinated			** < 0.001**			** < 0.001**
Yes	r(1)	r(1)		r(1)	r(1)	
No	12.7	8.1–19.9		10.8	6.7–17.5	
Multigenerational household	r(1)	r(1)	**0.002**	r(1)	r(1)	0.359
Yes	1.6	1.19–2.18		1.2	0.81–1.78	
No						
Dialysis via tunneled catheter	r(1)	r(1)	**0.022**	r(1)	r(1)	0.231
No	1.99	1.1–3.6		1.55	0.76–3.16	
Yes						
Flu shot received			**0.009**			0.356
Yes	r(1)	r(1)		r(1)	r(1)	
No	1.78	1.15–2.73		0.77	0.45–1.34	
Flu shot planned			** < 0.001**			** < 0.001**
Yes	r(1)	r(1)		r(1)	r(1)	
No/have not decided	4.47	3.02–6.63		4.16	2.6–6.8	

Notes: r(1) = reference category. COR = crude odds ratio. AOR = adjusted odds ratio. CI = confidence interval. The test of significance is binary logistic regression

## Discussion

Convincing the Egyptian population to receive the COVID-19 vaccine has already been a challenging process. Despite good public knowledge and awareness about COVID-19 severity and vaccine safety [22], hesitancy or refusal to take the vaccine has been present among a large portion of Egyptian citizens, even medical students [23] and healthcare workers [24, 25]. Patients on maintenance HD have been shown to have diminished and waning humoral responses after COVID-19 vaccination [26], which improved after a third [27] or a fourth booster dose [28]. For Egyptian patients on maintenance HD, a previous study showed that about 40% of patients were hesitant or resistant to taking the COVID-19 vaccine [14]. In this context, our study was conducted to identify the willingness of Egyptian HD patients to receive a booster dose of the COVID-19 vaccine. The study was conducted after the approval of booster doses in Egypt.

The present study shows male predominance (58.9%) among our surveyed HD patients. This is in agreement with the latest published statistics from the Egyptian Renal Data System (ERDS) which shows a 58.7% male predominance in the Egyptian HD population [29]. The majority of surveyed individuals in the present study were unemployed highlighting the problem of unemployment in the HD population. This is again in agreement with the ERDS which stated that around 81.11% of Egyptian HD patients are either unemployed, retired, housewives, or students [29].

Approximately 57% of surveyed individuals lived in multigenerational housing, which represents an independent risk factor for COVID-19 infection [30]. While dialysis represents a state of compromised immune system [31], comorbid conditions might aggravate the immunosuppression state. A minority of participants in the present study are affected by various comorbidities such as diabetes, previous kidney transplantation, a history of receiving immunosuppressive treatment in the previous 12 months, and dialysis through a tunneled catheter. On the other hand, the majority of surveyed individuals had a dialysis vintage of more than 3 years which represents an additional risk factor for COVID-19-associated acute respiratory distress syndrome and death [32].

Only about one-quarter of the participants in the present study reportedly became infected with SARS-CoV-2. This could be partly explained by the fact that some patients might have had asymptomatic COVID-19 infection as reported in previous studies [33–35]. Another explanation is the presence of a high COVID-19 vaccination rate among studied individuals (more than two-thirds), which is supported by the finding that the majority of patients had been infected with SARS-CoV-2 prior to undergoing vaccination.

Vaccination practice, hesitancy, and acceptance are influenced by the source of information [36], especially nowadays with social media disseminating misinformation [37]. Participants of this survey mostly received information about the COVID-19 vaccine from dialysis staff and less often from the treating physician and social media. This probably helped achieve a high vaccination rate with nearly 70% of patients vaccinated.

As the Sinopharm vaccine against COVID-19 was the first vaccine to be approved in Egypt, it was the most commonly received one by participants of this survey with nearly one-third of surveyed individuals receiving the full vaccination schedule (2 doses of Sinopharm vaccine). Patients reported many reasons for not receiving the vaccine at all or not completing the full schedule. Reasons reported by the patients were classified into 5 main categories; their confidence (which represented 50%), complacency, convenience or constraints, risk calculation and collective responsibility for protection of others through herd immunity [38].

The current study highlights an ongoing dilemma encountered since the beginning of the COVID-19 pandemic, it reflects frustration and indecisiveness regarding COVID-19 vaccines and the booster dose. About half of the participants (49.3%) exhibited their willingness to receive the booster dose of the COVID-19 vaccine. The majority of booster-hesitant individuals (44.9%) considered the booster dose unnecessary, while surprisingly only a minority were booster-hesitant owing to the side effects experienced after previous COVID-19 vaccine doses. This is in agreement with the Algerians [39] but contradicts the Polish population study [21]. A possible race or ethnic explanation for this contradiction may be raised, however, it is not clear whether race/ethnicity could explain differences in vaccine hesitancy among various populations. In fact, vaccine hesitancy differed among several racial and ethnic groups in the United States and United Kingdom in a recently published comparative study[40].

Although results obtained from surveys in developed countries were encouraging, with rates of willingness to receive vaccine boosters varying from 61.8 to 95.5% in the USA, Poland, Czech Republic, Germany, Japan, China, and Denmark [20, 21, 41–47], it seems that Egyptians still have a high degree of hesitancy to a booster dose. This is similar to rates of primary COVID-19 vaccination hesitancy documented in previous Egyptian surveys which showed a willingness rate of 25% in the general Egyptian population [48] and 58.3% in maintenance HD patients [14]. In the east, other Arab countries also showed low rates of booster acceptance accounting for 55% in Saudi Arabia [49], 51.6% in Algeria [39], and 39% in Jordan [50].

Indeed, these dissimilarities among countries regarding COVID-19 vaccine hesitancy could be ascribed to differences in sociodemographic and anamnestic characteristics, human behavior, sources of information about vaccines, control of rumors and misinformation, trust in the health care systems, available vaccines, uncertainties about vaccine side effects, and belief in vaccine benefits. Surprisingly, the results of this study indicate that not only differences in the rate of booster hesitancy are evident among countries but also within the same country. Residents of Alexandria were more likely to be booster hesitant than their counterparts in other governorates included in this survey, a finding that could be attributed to the high frequency of patients without full primary vaccination against COVID-19 in Alexandria (∼47%) compared with Dakahlia and Fayoum (∼13% and 24%, respectively). Similarly, urban residents constituted more than two-thirds of the not fully vaccinated participants.

The present study showed that various social and demographic factors are significantly associated with vaccine booster hesitancy. Regarding gender, females were significantly more booster-dose hesitant than males (OR: 1.47; 95% CI 1.09–2). This finding is in accordance with findings from other studies about initial vaccination hesitancy [48, 51]. This could be explained, for females in the childbearing period, by persistent false messaging on social media that COVID-19 vaccines may cause infertility in females or birth defects [52, 53].

Regarding the age of the participants in the present study, older participants were more likely to accept vaccine boosters than younger ones. This finding seems to match other studies that were carried out addressing the initial COVID-19 vaccination not only in the dialysis population [13, 54] but also in the general population [51]. Also, studies of booster hesitancy showed the same finding [21, 39].

Concerning marital status, single individuals who had never married were more likely to be booster-dose hesitant than married, divorced, or widowed individuals (OR 3.4; 95% CI 2.3–5.2). This is in agreement with a study carried out in Saudi Arabia which demonstrated that married individuals were more likely to accept vaccination [55].

Educational level has been a matter of debate as regards its correlation with vaccine hesitancy, with most studies reporting an inverse relationship between education and vaccine hesitancy [13, 20, 48, 51], while one study showed a positive correlation [39]. Nevertheless, other studies showed no association between vaccine hesitancy and education [14, 21, 54] which is in accordance with the results of the present study. Similarly, working status, previous COVID-19 infection, not receiving vaccine information, diabetes mellitus, prior history of kidney transplantation, dialysis vintage, and having a family member infected with, or who died of, COVID-19 were not associated with booster hesitancy in these study participants.

A striking finding in multivariate analysis in the current study is that participants who were already fully vaccinated against COVID-19 were nearly eleven times more likely to accept booster doses. This suggests that acceptance of primary vaccination is strongly associated with willingness to accept a booster dose once recommended. This finding is similar to that found in adult Americans [20].

Concordance between the answers on the influenza vaccine and the COVID-19 vaccine booster dose deserves further discussion: although, as expected, not having received the influenza vaccine was associated with a significantly higher likelihood of COVID-19 vaccine booster-dose hesitancy (OR of 4.47 for COVID-19 vaccine booster-dose hesitancy in the univariate analysis), the prevalence of influenza vaccine hesitancy was greater compared with COVID-19 vaccine booster-dose hesitancy (∼75% versus ∼50%). Interestingly, those who were not planning or who had not yet decided whether to receive the influenza vaccine were more likely to be COVID-19 booster-hesitant in the multivariate analysis. This could be attributed to a lower perceived severity of seasonal influenza than COVID-19 by patients on dialysis [56] or lack of awareness of the importance of annual influenza vaccination [57].

The current study demonstrates that the participants’ preferences for a specific COVID-19 vaccine to be used as a booster dose did not necessarily match the previously administered vaccine. Surprisingly, nearly half of the participants willing to receive a COVID-19 booster dose did not specify any particular vaccine preference. They did not choose a specific vaccination or make a decision at the time of the survey. This finding is similar to that found in the Polish population, of whom ∼55% did not know which vaccine to receive as a booster dose or showed no preference for a specific vaccine. Although the highest level of vaccine agreement in the present study was seen for AstraZeneca, a low level of agreement was observed in surveyed participants in Poland and the authors attributed this finding to safety concerns and lower degree of trust in this vaccine perceived by the Polish population [21]. On the other hand, another study in Algeria showed that vaccinated people are more likely to prefer a booster dose from the same vaccine manufacturer [39].

This study provides significant insight into factors associated with COVID-19 vaccine booster hesitancy among Egyptian HD patients. To our knowledge, this is the first study to explore this issue. A notable strength of the study is that it is multicenter and the sample is representative of HD patients in three Egyptian Governorates. The questionnaire was completed by professional health care workers through face-to-face interviews, not web-based as in the case of most published literature, eliminating the idea of the presence of missing questions or data. Nevertheless, the study has several limitations. First, it is a cross-sectional study taking a snapshot of HD patients' willingness to take the booster vaccine, while in reality, individual attitudes are dynamic and evolving, and the intention to vaccinate is generally context-dependent. Second, as is the case for all cross-sectional studies, causality cannot be inferred from this design.

Healthcare authorities in Egypt should be aware of the magnitude of the vaccine hesitancy problem among hemodialysis patients and act accordingly to resolve such a problem. The results of the current study could be used to identify solutions to the vaccine hesitancy problem. Focusing on the younger generations, female gender, unmarried, and unvaccinated patients to raise their awareness and fight back against false information is mandatory to decrease vaccine hesitancy. Since healthcare workers played a critical role in the delivery of information to the participants of the present study, they should raise awareness regarding the general attitude toward vaccination focusing on the importance of vaccination and the safety and efficacy of the available vaccines. Another effective strategy for reducing booster hesitancy, particularly for in-center HD patients, is the provision of COVID-19 vaccination as a component of routine in-center care which significantly reduced the odds of vaccine hesitancy in South Africa [58].

## Conclusion

COVID-19 booster-dose hesitancy is highly prevalent among hemodialysis patients in Egypt. Health authorities should invest in health promotion to disseminate the right medical information to improve vaccine uptake.

## Supplementary Information

Below is the link to the electronic supplementary material.

Supplementary file1 (DOCX 24 KB)

## Data Availability

The corresponding author will provide the datasets used and/or analyzed during the current work upon reasonable request.
