# Supplementary material for: Egyptian hemodialysis patients' willingness to receive the COVID-19 vaccine booster dose: a multicenter survey
Source: J Nephrol. 2023 Mar 2;36(5):1329–40. doi: 10.1007/s40620-023-01586-z (PMC9979119; doi:10.1007/s40620-023-01586-z)
Supplement: Supplementary file 1 — Supplementary file1 (DOCX 24 KB) [file 40620_2023_1586_MOESM1_ESM.docx]

| **Demographic Data** | | |
| --- | --- | --- |
| **1** | **Age** | - 18-30 years - 30-50 years - >50 years |
| **2** | **Gender** | - Male - Female |
| **3** | **Marital Status** | - Single (never married) - Married - Divorced/Separated/Widowed |
| **4** | **Level of Education** | - Primary - Secondary - Vocational - Tertiary |
| **5** | **Employment status** | - Employed - Retired - Out of work - Still a student |
| **6** | **Residence** | - Rural - Urban |
| **7** | **Government** |  |
| **COVID-19 Vaccine** | | |
| **8** | **From where do you receive information about the COVID-19 vaccines (Select all that apply)?** | - Dialysis staff (nurses or technicians) - My kidney doctor - Other patients on dialysis - Friends and family - TV news - Newspapers - Social media (Facebook, Twitter, Instagram, etc) - I have not received any information about COVID-19 vaccines - Other |
| **9** | **Have you been fully vaccinated against COVID-19 (received 2 doses of BioNTech-Pfizer, Moderna or AstraZeneca, Sinopharm, Sinovac or a single dose of Janssen/Johnson&Johnson)?** If the answer is No, please mention the reason | - Yes - No |
| **10** | **Which COVID-19 vaccine have you been vaccinated with?** | - BioNTech-Pfizer (2 doses) - Moderna (2 doses) - AstraZeneca (2 doses) - Janssen/Johnson&Johnson (1 dose) - Sinopharm (2 doses) - Sinovac |
| **11** | **Please evaluate the severity of side effects that occurred after receiving your COVID-19 vaccine** | - No side effects/negligible side effects - Medium severity - very high severity |
| **12** | **Please evaluate the level of fear accompanying the side effects that occurred after receiving your COVID-19 vaccine** | - no fear/very low level of fear - medium fear - very high fear |
| **13** | **Are you willing to receive the potential additional dose of the COVID-19 vaccine if it would be made available?** | - Yes - No - Don’t know |
| **14** | **(If the answer "yes" in question #13) Which vaccine would you like to receive as the additional COVID-19 vaccine dose?** | - It doesn't matter - I don't know - BioNTech/Pfizer - Moderna - AstraZeneca - Janssen/Johnson&Johnson - Sinopharm - Sinovac |
| **15** | **(If the answer "yes" in question #13) Please evaluate the level of fear associated with receiving the potential additional dose of the COVID-19 vaccine?** | - No fear/very low level of fear - Medium fear - Very high fear |
| **16** | **(If the answer "no" in question #13) Why are you not willing to receive the additional dose of the COVID-19 vaccine dose?** | - I don't think it is necessary - Due to safety concerns - Due to side effects after previous doses of COVID-19 vaccine |
| **17** | **Have you been infected with SARS-CoV-2?** | - No - Yes, prior to vaccination - Yes, between 1st and 2nd dose of vaccine (for those vaccinated with BioNTech/Pfizer, Moderna or AstraZeneca vaccines) - Yes, after receiving all required doses (two doses of BioNTech/Pfizer, Moderna or AstraZeneca vaccines or a single dose of Janssen/Johnson&Johnson vaccine) |
| **Health & Family** | | |
| **18** | **Do you have, or have you ever had, diabetes?** | - Yes - No - Don’t know |
| **19** | **Have you had a kidney transplant?** | - Yes - No |
| **20** | **In the past 12 months have you been on medications that may lower your ability to fight infections (e.g., an immunosuppressant)?** | - Yes - No - Don’t know |
| **21** | **Do you do dialysis via a tunneled catheter?** | - Yes - No |
| **22** | **How many years have you been on dialysis?** | - less than 1 year - 1 to 3 years - more than 3 years |
| **23** | **Has a close family member (parent, partner, brother or sister, child, grandparent or grandchild) or a close acquaintance (a person you see or interact with weekly) been sick with COVID-19?** | - Yes - No |
| **24** | **Has a close family member or a close acquaintance died of COVID-19?** | - Yes - No |
| **25** | **Do you live in a multigenerational household (household with elderly adults such as grandparents, and children)?** | - Yes - No |
| **26** | **Have you gotten the flu shot?** | - Yes - No |
| **27** | **Do you plan to get the flu shot this year 2022/2023?** | - Yes - No - Have not decided |
